# The Best in CytoJournal: 2005

**DOI:** 10.1186/1742-6413-3-21

**Published:** 2006-09-13

**Authors:** Michael B Cohen

**Affiliations:** 1 Department of Pathology, 200 Hawkins Drive, C670 GH, The University of Iowa, Iowa City, IA, USA

## Abstract

CytoJournal opened its doors in late 2004 and 2005 was the first full year of publication. In order to celebrate this event as well as recognize the contributing authors works, CytoJournal has initiated a Best Article of the Year, in this case 2005. This year's Best of CytoJournal: 2005 is an excellent review of thyroid FNAB.

## Background

This is an exciting time in peer-reviewed publication. The establishment of BioMed Central and its on-line journals, including CytoJournal, has opened up a new avenue for rapid publication and open access [1]. In order to help with further enhancing CytoJournal, the journal Editors asked me to assemble a small group of cytopathologists from the international advisory board to identify the best article from all the articles published through 2005 [2-28]. On behalf of this ad hoc committee, I am pleased to announce to the readers of CytoJournal that the article by Drs. Nguyen, Lee, Ginsberg, Wragg, and Bilodeau, Fine-needle aspiration biopsy of the thyroid: an overview, was chosen [2]. Notably, it is also one of the 'highly accessed' articles [29].

It has been estimated that about 5% of the general population has a palpable thyroid nodule [2]. Thyroid fine needle aspiration biopsy (FNAB) is a very useful way of evaluating and managing such nodules. In fact, it is often the initial triage point. The review by Drs. Nguyen, et al. provides a succinct discussion of the cytologic features that can be encountered by cytologists in evaluating thyroid FNABs, including the utility of ancillary studies.

In closing, we congratulate the authors on their article and hope that at the same time this will interest other authors in submitting their articles to this journal.
